# Economic and epidemiologic impact of guidelines for early ART initiation irrespective of CD4 count in Spain

**DOI:** 10.1371/journal.pone.0206755

**Published:** 2018-11-05

**Authors:** Parastu Kasaie, Matthew Radford, Sunaina Kapoor, Younghee Jung, Beatriz Hernandez Novoa, David Dowdy, Maunank Shah

**Affiliations:** 1 Johns Hopkins Bloomberg School of Public Health, Baltimore, Maryland, United States of America; 2 ViiV Healthcare Inc, Brentford, Middlesex, United Kingdom; 3 Johns Hopkins School of Medicine, Baltimore, Maryland, United States of America; Azienda Ospedaliera Universitaria di Perugia, ITALY

## Abstract

**Introduction:**

Emerging data suggest that early antiretroviral therapy (ART) could reduce serious AIDS and non-AIDS events and deaths but could also increase costs. In January 2016, the Spanish guidelines were updated to recommend ART at any CD4 count. However, the epidemiologic and economic impacts of early ART initiation in Spain remain unclear.

**Methods:**

The Johns Hopkins HIV Economic-Epidemiologic Mathematical Model (JHEEM) was utilized to estimate costs, transmissions, and outcomes in Spain over 20 years. We compared implementation of guidelines for early ART initiation to a counterfactual scenario deferring ART until CD4-counts fall below 350 cells/mm^3^. We additionally studied the impact of early ART initiation in combination with improvements to HIV screening, care linkage and engagement.

**Results:**

Early ART initiation (irrespective of CD4-count) is expected to avert 20,100 [95% Uncertainty Range (UR) 11,100–83,000] new HIV cases over the next two decades compared to delayed ART (28% reduction), at an incremental health system cost of €1.05 billion [€0.66 – €1.63] billion, and an incremental cost-effectiveness ratio (ICER) of €29,700 [€13,700 – €41,200] per QALY gained. Projected ICERs declined further over longer time horizon; e.g., an ICER of €12,691 over 30 years. Furthermore, the impact of early ART initiation was potentiated by improved HIV screening among high-risk individuals, averting an estimated 41,600 [23,200–172,200] HIV infections (a 58% decline) compared to delayed ART.

**Conclusions:**

Recommendations for ART initiation irrespective of CD4-counts are cost-effective and could avert > 30% of new cases in Spain. Improving HIV diagnosis can amplify this impact.

## Introduction

Advances in HIV treatment and prevention programs have decreased the worldwide annual incidence of HIV infections to 2.1 million cases per year [1]. Global increases in the number of people receiving antiretroviral therapy (ART) over the last decade have further led to a decrease in the number of people dying from HIV-related causes by 45% compared to HIV mortality in 2005 [2]. This has been in part due to the evolution of worldwide guidelines which in 2002, recommended ART initiation in persons living with HIV (PLWH) with CD4 cell counts less than 200 cells per cubic milliliter [3], and now recommend initiation of ART irrespective of CD4 count [4]. This progression towards earlier ART initiation has been based on clinical trials highlighting the efficacy of early ART initiation [5, 6]. ART initiation earlier in the natural history of HIV infection has both public health benefits attributable to reduced transmission and individual level benefits of improved health. The INSIGHT-START study, for example, found that early ART (initiation at CD4 >500 cells/mm^3^) reduced the risk of AIDS-related events, non-AIDS related events, and all-cause mortality compared to late ART (initiation at CD4 <350 cells/mm^3^) by 57% [95% CI: 38–70%][5]. Furthermore, early ART initiation has been found to be more effective in preventing HIV transmission compared to late initiation due to earlier sustained viral suppression [7].

Spain, with an estimated population of 150,000 people living with HIV (PLWH), adopted policies for early ART initiation in 2016 [8, 9]. Despite updated recommendations, however, the epidemiologic and economic impacts of early ART initiation, including impacts on AIDS-related events, mortality, transmission, and costs, have not been previously studied. Furthermore, as these new policies are scaled up, health system costs related to ART utilization are likely to increase. We thus sought to evaluate the cost-effectiveness and population-level impact of implementing early ART initiation policies irrespective of CD4 count in Spain compared to delayed initiation at lower CD4 thresholds (less than 350 cells per cubic milliliter). Moreover, we sought to evaluate how current levels of HIV care engagement in Spain (i.e. the HIV care continuum) may impact new policies towards timing of ART initiation.

## Methods

The Johns Hopkins HIV Economic-Epidemiologic Mathematical Model (JHEEM) was utilized to assess the epidemiologic and economic impacts of early ART initiation at any CD4 count in PLWH compared to delayed treatment in which ART is deferred until CD4 count falls below 350 cells/mm^3^. The target population was PLWH in Spain, and the cost analysis was conducted from the health system perspective. Primary outcomes included health system costs, serious clinical events (AIDS and non-AIDS events), incident cases, deaths, and Quality Adjusted Life-Years (QALYs) gained over a time horizon of 20 years (year 2017 to 2037). Secondary analysis explored longer time horizons and the modulating effect of improved HIV screening, linkage and retention in care on the impact of early ART initiation.

### Simulation model

The JHEEM model [10, 11] is a compartmental model of the HIV epidemic that incorporates transmission dynamics, disease progression and health system engagement. We updated the model to represent the population of Spanish adults (age 15 to 83), stratified by sex (male or female), HIV infection status, and HIV risk practices, as well as transmission routes including heterosexuals (age-stratified), men who have sex with men (MSM), and people who inject drugs (PWID). HIV transmission is modeled through sexual contact among heterosexual population or MSM, and needle sharing among PWID. Risk of HIV transmission is determined by frequency of sexual interactions and needle sharing within and across risk groups, stage of HIV infection (e.g. higher transmission risk during acute HIV) and ART usage. Upon infection with HIV, individuals progress through a series of disease stages (stratified by CD4 count) based on rates of HIV progression and engagement with the care continuum, including: 1) unaware of HIV status, 2) diagnosed but not in care, 3) in care but not on ART, 4) on ART but not virologically suppressed, and 5) on ART and virologically suppressed. The model is developed as a system of ordinary differential equations [10, 11]. Additional model details are found in Section A of the S1 File.

### Model parameters and simulation calibration

**Fig 1:**
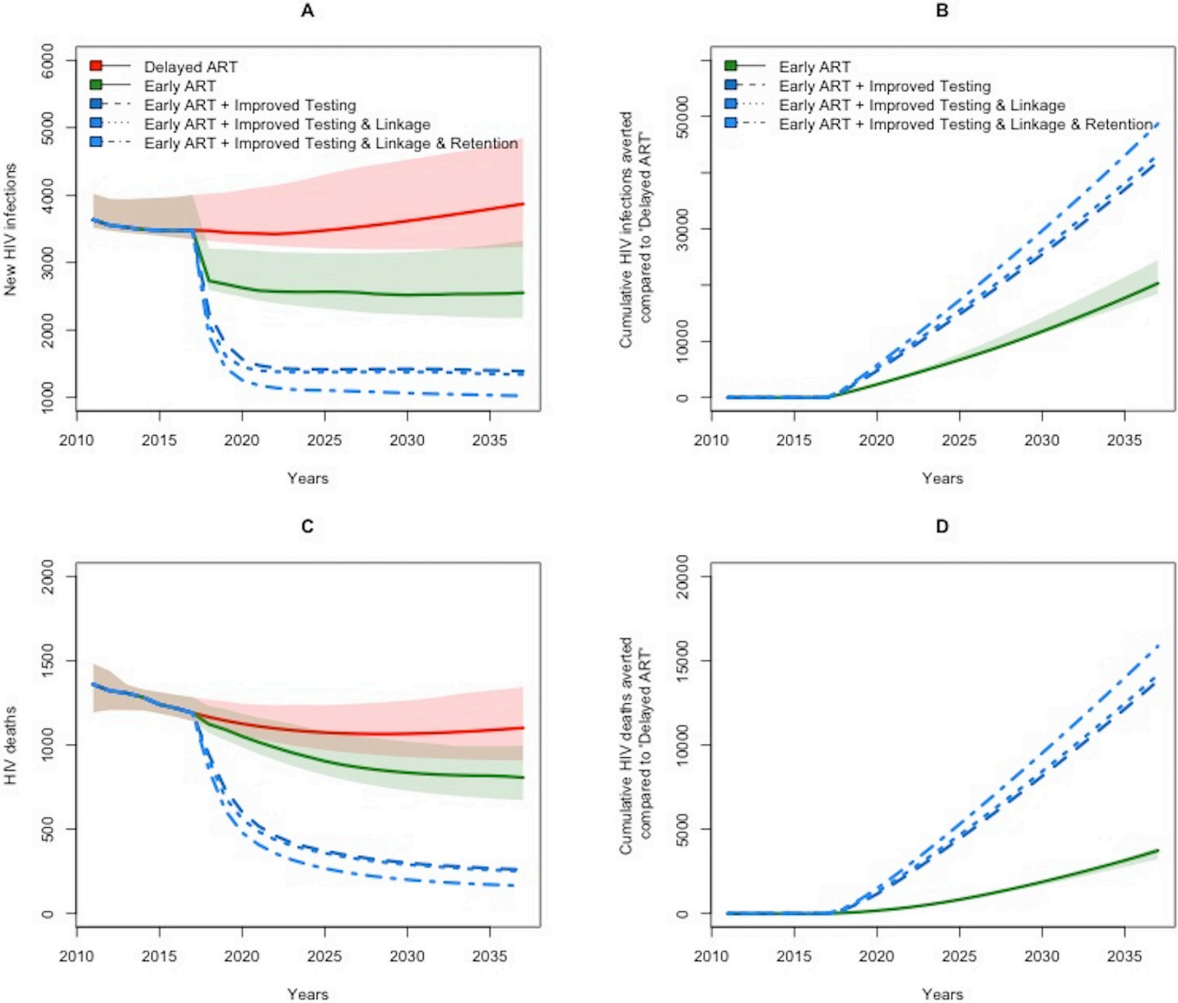
**Projected new HIV infections (A), cumulative HIV infections averted (B) HIV deaths (C), and cumulative deaths averted (D) in Spain from 2017 to 2037 under early versus delayed ART.** All lines represent the median values of simulations, comparing scenarios representing delayed ART initiation at CD4 counts of <350 cells/mm^3^(solid red line in Panels A&C) against early ART initiation, both without (solid green line) and with additional improvements to the HIV care continuum (dashed blue lines). The shaded areas represent the corresponding interquartile uncertainty ranges (not shown for the additional improvement scenarios due to overlap).

Key epidemiologic and HIV natural history parameters [10, 11] were derived from the literature (Table 1). All presented parameters have a fixed value in the main analysis, and the ranges are used for sensitivity and probabilistic uncertainty analysis. Additional parameters relating to HIV transmission (e.g., partnerships per year) and care-continuum parameters (e.g., rate of HIV screening, linkage to care, and annual retention in care) were calibrated to reflect observed estimates of HIV prevalence, incidence and death in Spain [12–16]. To calibrate the model, we modeled a baseline scenario representing delayed ART initiation at lower CD4 thresholds (less than 350 cells per cubic milliliter), and used a Bayesian melding estimation approach for calibrating the simulation and making future projections [17–19]. For this purpose, we first brought the model to an equilibrium representative of the size of Spain’s HIV epidemic in 2010. We then estimated the prior distributions of unknown transmission and care-engagement parameters in the model, and drew randomly from these distributions to generate a set of initial simulations. After comparing the simulation outcomes with selected calibration targets (e.g., HIV incidence and death in Spain from 2011 to 2015), we computed a pseudo-likelihood associated with each simulation reflecting that simulation’s fit to the calibration data and then re-sampled 1 million simulations (with replacement) with a probability reflecting each simulation’s pseudo-likelihood. The re-sampled simulations then formed the posterior distribution of simulations, from which we calculated the quantities of interest (e.g., calibrated HIV incidence from 2011 to 2015 and projected levels from 2017 to 2037 at baseline as shown in Fig 1A, red line). Further details regarding the calibration procedure are provided in Section B of the S1 File.

**Table 1:** Key model input parameters. Event rates and ART effects were based on START Trial; we used literature estimates from Spain for costs of various AIDS and non-AIDS events, and utilized a weighted average based on specific event rates in START trial.

Parameter	Fixed value	Range	References
**Costs**			
ART (average of GESIDA 2017 first-line)	€ 8,600	€6,435 – €22,180	[9, 20–22]
HIV test	€ 19	€10 – €50	[23]
Genotype	€ 384	192–576	[21]
Viral Load test	€ 100	€20 – €150	[20, 21, 24]
CD4 count test	€ 50	25–75	[25–27]
Office visit	€ 104	€50 – €250	[20–22]
Cost of AIDS events (weighted average of TB, lymphoma, Kaposi Sarcoma, pneumocystis)	€ 19,000	€9,500 – €100,000	[28–32]
Cost of non-AIDS events (weighted average of MI, CVA, other malignancies)	€ 8,000	€5,000 – €17,000	[28–32]
**AIDS/Non-AIDS events**			
AIDS events rate per year (increased for lower CD4 counts)	0.0072	0.005–0.01	[5, 33, 34]
Non-AIDS event rate per year (increased for lower CD4)	0.0067	0.005–0.01	[30–32]
Hazard ratio with ART (AIDS events)	0.28	0.015–0.5	[5, 33, 34]
Hazard ratio with ART (non-AIDS events)	0.61	0.38–0.97	
**Utility Weights**			
Uninfected	1	—	[35, 36]
Acute HIV	0.84	0.8–0.9	[35, 36]
HIV Unsuppressed CD4>350	0.94	0.9–0.99	[35, 36]
HIV Unsuppressed CD4 200–350	0.84	0.8–0.99	[35, 36]
HIV/AIDS Unsuppressed CD4 < 200	0.7	0.5–0.9	[35, 36]
ART usage	0.96	0.94–1	[35, 36]
Reduction in utility losses with viral suppression	50%	0%– 90%	Assumption
**Natural History and Transmission**			
Duration of Early Infection: CD4>350	6.5 years	3–10 years	[37–39]
Duration of Late Infection: CD4 200–350	2.5 years	1–5 years	[37, 38]
Duration of AIDS CD4 < 200	2 years	1–5 years	[39–44]
Relative risk reduction of transmission with ART	95%	80–99.5%	[45–48]
Relative risk reduction of AIDS death with ART	95%	50%– 98%	[5, 44, 49]

### Simulation scenarios

We developed a series of counterfactual scenarios to model the impact of early ART initiation (irrespective of CD4 count) compared to delayed initiation at lower CD4 thresholds (less than 350 cells per cubic milliliter). As a secondary analysis, we studied early ART initiation in combination with improved HIV testing for high-risk individuals (i.e., modeled as annual HIV screening among young individuals, MSM and PWID), improved linkage (i.e., modeled as 100% linkage to care after HIV diagnosis) and improved retention in care (i.e., modeled as a reduction in rate of disengagement and average delay before re-engagement in care)- see Section C of the S1 File. Interventions were modeled as an immediate scale-up in the rate of ART initiation (and other relevant parameters) at year 2017 and were modeled over the next two decades (to 2037). Additional analyses were performed to estimate the longer-term impact of interventions at 30 and 40 years after implementation.

### Epidemiological outcomes

The main outcomes of interest included the number of incident HIV cases, AIDS events (defined as death from AIDS or any AIDS-defining event) and non-AIDS events (defined as death from causes other than AIDS including cardiovascular, renal, liver disease, non–AIDS-defining cancer, and any death)[5], comparing the scenarios of early versus delayed ART initiation. All outcomes are presented as the median value and the ninety-five percent uncertainty ranges (the 2.5th and 97.5th percentiles of simulated outcomes) from the posterior distribution of simulations, as described above.

### Economic impacts and costs

Each model compartment was assigned a health utility, associated with the corresponding stage of HIV. We assigned unit costs both to specific transitions between compartments (e.g., initiation of ART) and to person-time spent in specific compartments (e.g., person-years of ART use). Total healthcare costs and QALYs were then calculated as the sum of all unit costs and of (person-time) *(health utility) across all compartments over time, respectively. Costs were reported in terms of 2017 Euros (EUR) (either evaluated in 2017 or converted from previous years to reflect value in 2017 using appropriate conversion rates [50]); future costs and QALYs were discounted at a rate of 3% annually [51]. Costs, utility weights and other key model parameters are shown in Table 1. Cost-effectiveness was measured as the Incremental Cost-Effectiveness Ratio (ICER) and was assessed based on a Willingness-To-Pay (WTP) threshold of €30,000 per QALY averted which has been previously used in Spain [52–54].

### Sensitivity and uncertainty analysis

We performed one-way sensitivity analysis to evaluate the influence of each parameter individually on primary outcomes comparing early vs. delayed ART initiation (comparing simulations containing the highest and lowest deciles of each parameter value) on selected outcomes. Additionally, we conducted probabilistic uncertainty analyses by performing a large number of simulations simultaneously varying all model parameter within specified ranges (see selected ranges in Table 1). This analysis was utilized to estimate the probability of cost-effectiveness at varying WTP thresholds and was represented in a cost-effectiveness acceptability curve.

### Ethical considerations

This economic evaluation was evaluated by the ethics committee of the Johns Hopkins University, Baltimore, Maryland, USA, and was deemed exempt, as it did not constitute human subjects research.

## Results

### Epidemiologic impact

In the counterfactual scenario that ART initiation was delayed until a CD4 count of 350 cells/mm^3^ through 2037 in Spain, we estimated a median of 71,400 [95% Uncertainty Range (UR) 50,800–257,700] incident HIV infections between 2017 and 2037. By contrast, immediate and broad-scale implementation of current Spanish guidelines for ART initiation at any CD4 count was projected to avert 20,100 [11,100–83,000] of these 71,400 cases (a 28% reduction) (Fig 1). When examined over longer time horizons, we found that these epidemiologic impacts are amplified. Specifically, we estimated that early ART initiation could avert 33,600 [16,800–62,600] of 104,900 [77,800–193,100] infections over 30 years and 44,500 [22,700–100,300] of 148,100 [102,700–267,400] infections over 40 years. (For more information, see Section D.2 of the S1 File).

We additionally estimated substantial declines in HIV related morbidity and mortality, with an estimated 9,000 [6,300–26,400] AIDS- and non-AIDS events averted (a 15% reduction), and 3,800 [2,100–11,400] early deaths averted (a 17% reduction). These benefits translated to over 33,800 [21,800–92,900] additional QALYs gained over 20 years (with discounting). Overall, despite these projected benefits, our results suggest only modest improvements in the percent of PLWH that are virally suppressed by 2037 (8% [5%– 14%] improvement). When considering current levels of diagnosis and care engagement; current data suggests that the primary gap in the care continuum in Spain is the undiagnosed fraction, whereas over 60–80% of those in care are on ART and suppressed [55–60].

In a secondary analysis, we examined the degree to which improvements in the HIV care continuum could modulate the impact of early ART initiation. If individuals with high-risk practices (younger heterosexuals, MSM and PWID) were screened annually for HIV, we projected a 58% reduction in new HIV cases (averting 41,600 [23,200–172,200] cases) when implementing guidelines for early ART irrespective of CD4 count (Table 2). Further improvements to rate of linkage and retention in HIV care achieved only modest additional improvements; for example, we estimated that early ART and annual screening, combined with improved linkage to care, would avert 42,900 [24,300–179,400] new infections. Further improvements in retention in care increased this estimated impact to 47,800 [30,200–197,100] infections averted over 20 years). For a complete list of outcomes, see Section D.1 of the S1 File.

**Table 2:** Costs and effectiveness of selected ART initiation scenarios in Spain over the next 20 years. All outcomes represent the median [95% uncertainty ranges] across simulations. Future costs and QALY’s are discounted.

Scenario	HIV incidence	AIDS and non-AIDS Events	HIV Deaths	Health System Costs (billions)	QALY’s	ICER
**Delayed ART (CD4<350)**	71400 [50800–257700]	60000 [47200–131400]	21800 [15300–57800]	€19.55 [€17.19 – €23.81]		
**Early ART (Any CD4)**	51500 [33700–181100]	50600 [39400–107400]	18000 [12200–47300]	€20.63 [€18.16 – €25.15]		
**Δ Incremental (vs. delayed ART)**	20100 [83000–11100]	9100 [6300–26400]	3800 [11400–2100]	€1.05 [€0.66 – €1.63]	33800 [21700–92900]	€29700 [€13700 – €41200]
**Early ART+ Improved Testing**	29800 [16900–91200]	35800 [28700–69500]	7900 [4900–23000]	€22.96 [€20.56 – €27.41]		
**Δ Incremental (vs. delayed ART)**	41600 [172200–23200]	23100 [15700–67800]	13200 [37600–8000]	€3.38 [€2.64 – €4.28]	113900 [71700–297500]	€29500 [€12800 – €40600]

### Costs and cost-effectiveness

We estimated that early ART initiation would result in an incremental increase of €1.05 billion [€0.66 – €1.63] billion in (discounted) costs. This translated to a median incremental cost-effectiveness ratio (ICER) of €29,700 [€13,700 – €41,200] per (discounted) QALY gained over 20 years as represented in Table 2. Given challenges in interpreting absolute QALY’s (with future discounting), we present only the incremental QALY’s-gained comparing the Delayed ART scenario to early ART scenarios. The incremental costs of early ART declined further when projected over longer time horizons, and incremental cost-effectiveness likewise improves. We estimated an incremental cost of €0.84 billion [€0.40 – €1.31] billion, and incremental cost-effectiveness of €12,700 [€4,300 – €21,000] per QALY gained, over 30 years, and an incremental cost of €0.52 billion [€-0.67 (i.e. cost saving)– €1.10] billion and incremental cost-effectiveness of €5,200 [€-4,600 (i.e. cost saving)– €11,300] per QALY gained at 40 years.

In secondary analysis, we found that combining early ART initiation with annual testing of groups with high-risk practices would cost €3.38 billion [€2.64 – €4.28] billion more than delayed ART (without expanded testing) (inclusive of annual test costs, but not of ancillary programmatic costs to reach high-risk groups), but would achieve similar cost-effectiveness ratios as early ART alone, given the additional health benefits and averted transmissions in this scenario (€29,500 [€12,800 – €40,600] per QALY gained over a 20 year time horizon, Table 2).

**Fig 2:**
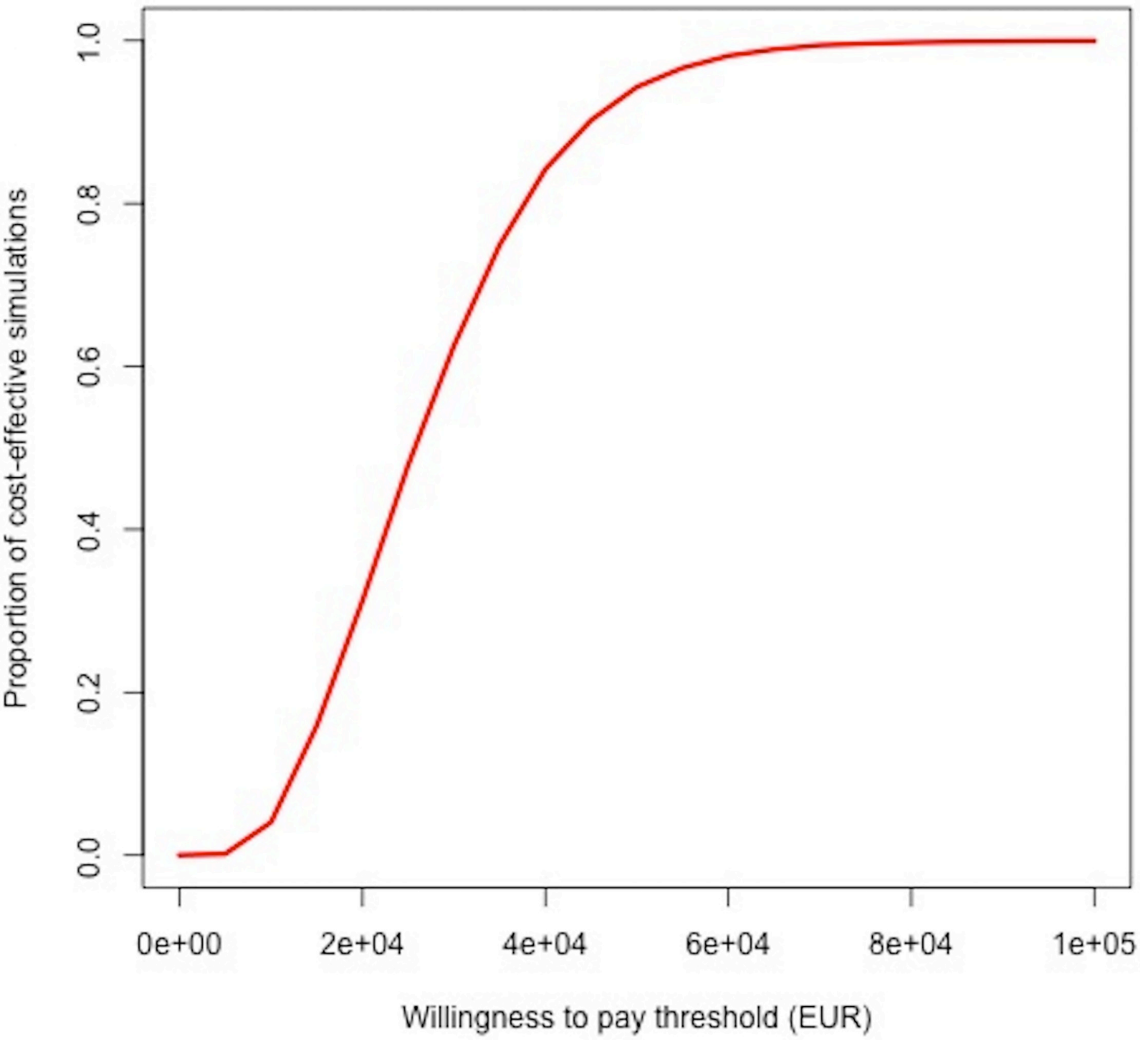
Cost-effectiveness acceptability curve for early (versus delayed) ART initiation. The y-axis represents the proportion of all simulations that were shown to be cost-effective in the probabilistic uncertainty analysis under different willingness-to-pay thresholds, expressed as 2017 euros per QALY gained (on the x-axis). At current proposed WTP thresholds of €30,000 per QALY averted, 62% of simulations are cost-effective (dashed blue lines). Increasing the WTP to €80,000 increases the proportion of cost-effective simulations to 99%.

We conducted a probabilistic uncertainty analysis in which all parameters were varied widely simultaneously in order to construct a cost-effectiveness acceptability curve at varying WTP thresholds (Fig 2). At current proposed WTP thresholds of €30,000 per QALY averted, 62% of simulations were cost-effective. Increasing the WTP to €80,000 as has been proposed in some European settings (or representing ~3x GDP per capita in Spain as proposed by WHO[61]) increased the proportion of cost-effective simulations to 99%.

### Sensitivity analysis

**Fig 3:**
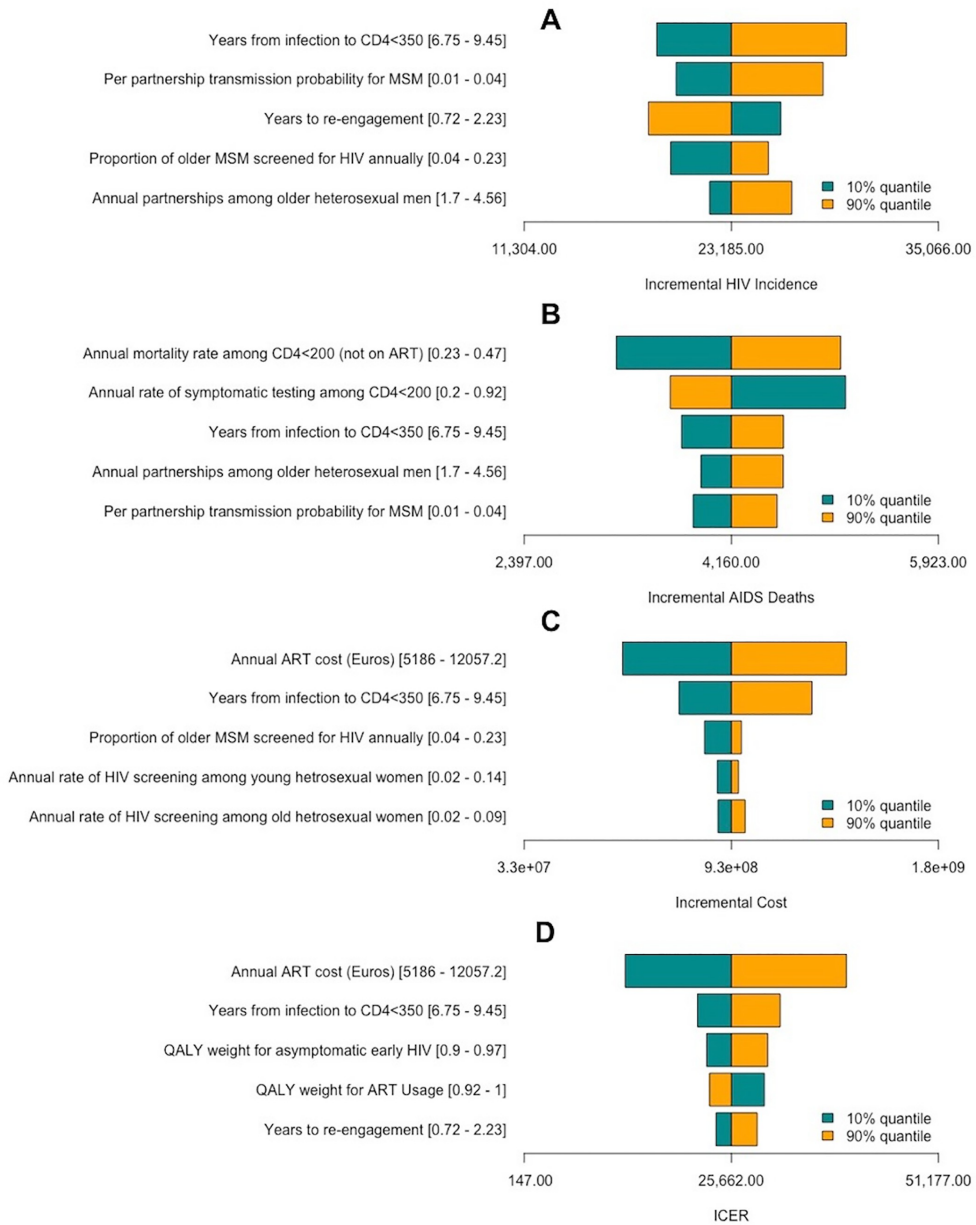
One-way sensitivity analysis of the incremental impact of early ART initiation compared to delayed ART initiation (CD4<350). Shown on the x-axis are the range of values for incremental HIV incidence (A), HIV deaths (B), costs (C) and cost-effectiveness (D) in the early ART initiation scenario compared to the delayed ART scenario. Shown on the y-axis are the five parameters [range of values] for which variation over their stated ranges resulted in the greater effect on each outcome. Yellow bars correspond to the median outcome value among the simulations containing the highest 10% of the parameter value, whereas green bars depict the median projected outcome among simulations containing the lowest 10% of the parameter value.

In one-way sensitivity analysis, we found that the ICER was most impacted by the cost of ART and ranged from €12,600 to €39,800 per QALY-gained for annual ART costs ranging €5,200 to €12,000 per year (Fig 3-Panel D). A similar pattern was observed for sensitivity of incremental healthcare costs to annual cost of ART and natural history of HIV progression to AIDS and death (Fig 3-Panel C). Incremental HIV incidence (i.e. comparing delayed and early ART scenarios) was most sensitive to variation in parameters pertaining to early HIV progression and rates of HIV transmission (Fig 3-Panel A). Further results are provided in Section E of the S1 File.

## Discussion

European and Spanish guidelines now recommend initiation of ART in PLWH irrespective of CD4 count, given that early ART initiation has been found in clinical research studies to reduce serious AIDS events, non-AIDS events, and HIV transmission[5, 6, 8, 9]. Increasing ART utilization may therefore improve clinical outcomes and reduce incidence, but is likely to increase costs associated with a longer duration of lifetime ART usage. We therefore sought to quantify the economic and epidemiologic impact of these new recommendations for early ART initiation compared to delayed treatment in Spain. The results of our model suggest that early ART initiation, compared to late ART initiation at CD4 count < 350 cells/mm3, may avert nearly 30% of new HIV cases over two decades, demonstrating the importance of ‘treatment as prevention’. Furthermore, early ART is likely to result in improved health outcomes for PLWH in AIDS events, non-AIDS events, and deaths avoided. Our results suggest that 16% of AIDS and non-AIDS events could be averted as a result of new recommendations for earlier ART initiation, compared to prior recommendations of delaying ART until lower CD4 thresholds. As such, early ART initiation has a high probability of being considered highly cost-effective in Spain.

Furthermore, we sought to examine how gaps in the HIV care continuum could modulate the new guidelines for earlier ART initiation. Currently, policy makers have established optimistic goals of “90-90-90” to achieve 90% of PLWH being aware of HIV status, 90% of PLWH receiving ART, and 90% of PLWH with sustained viral suppression by the year 2020[57, 62, 63]. Current HIV care continuum estimates suggest that Spain is short of reaching these targets, as nearly 20% of PLWH are unaware of their HIV status, 20–30% of diagnosed individuals have never received ART, and ultimately only 50%-70% of all PLWH have sustained viral suppression[55–60]. Overall, the primary gap in the care-continuum in Spain appears to be at the level of delayed diagnoses [55, 56, 58, 64]. We found that combining early ART with improved HIV screening (annual screening of groups with high-risk practices) could result in substantial additional gains in epidemiological impact, at similar cost-effectiveness ratios as early ART alone. This likely reflects the fact that screening remains a major gap in the HIV care continuum in Spain. If even more ambitious care continuum targets are achieved (annual high-risk screening with improved linkage and retention), then early ART initiation was projected to lead to ~86% of all PLWH virally suppressed (see Section D1 of the S1 File), and nearly 67% of new cases could be averted, compared to older guidelines for delayed ART initiation. These results highlight the necessity of combining early ART initiation with improvements in the HIV care continuum such as screening and linkage to care in order to achieve maximal economic and epidemiologic benefits. Our model results project that long-term viral suppression through earlier diagnosis and treatment have community level benefits with reductions in transmission allowing ‘treatment as prevention’ [7, 48].

It is important to understand the implications of our study results in the context of healthcare expenditures. Based on recent estimates, HIV/AIDS health care costs represent 1.25% of total healthcare expenditure in Spain [20]. There is therefore a need to consider the cost-effectiveness of new national guidelines and policy decisions. Prior work has sought to evaluate costs and cost-efficacy of various ART regimens in Spain and other European countries [21–23, 65–67]. Our work now builds on these efforts and demonstrates that, while likely to increase total health care costs (when examined over a 20-year period), early ART initiation represents excellent value for money (€29,700 [€13,700 – €41,200] per QALY gained) at current WTP thresholds. Our analyses suggest that these incremental costs are largely driven by the cost of ART as a larger proportion of individuals are expected to require ART (over longer periods of time) with the new recommendations for earlier ART initiation. However, our results also suggest that the impact of policy changes for earlier ART initiation (irrespective of CD4 count) are likely to yield accumulating benefits over time in terms of declining incremental costs and increasing health benefits (i.e. QALY’s-gained), with lower ICERs. When examining longer time horizons (30 or 40 years), our results suggest that the incremental costs (compared to the counter-factual scenario of delaying ART initiation) decline over time, and that early ART initiation becomes increasingly effective and cost-effective as longer time horizons are considered.

Our study has several important limitations. As with all economic models, there can be uncertainty in cost parameters. We utilized an extensive literature search to identify locally relevant cost inputs, and conducted sensitivity analysis where such data was limited. Moreover, we did not model specific ART regimens, but utilized average costs of currently recommended regimens in Spain [9]. To the extent that the population distribution of ART regimens is dominated by cheaper or more expensive specific regimens (or will change over time), our cost estimates may under or over-estimate true HIV associated health system expenditures. Nonetheless we explored wider ranges of average costs in sensitivity analysis, and found that early ART initiation has a high probability of being cost-effective (62%) at conservative WTP thresholds over 20 years. From a modeling perspective, our model is calibrated to available data on national-level HIV epidemiology. To the extent that there are subnational or regional variations in HIV incidence and care engagement, our results may over- or under-estimate the relative impact of improvements in local care-continua or transmission dynamics [56]. Furthermore, our modeling methodology makes simplifying assumptions about sexual or injection drug using networks (e.g., homogenous contact patterns with limited mixing) that can impact estimates; on the other hand, we utilized a Bayesian modeling approach to capture and explore the impact of such uncertainty more fully compared to alternative modeling approaches. Finally, our current analysis does not incorporate the potential role of pre-exposure prophylaxis (PrEP) or other future interventions for prevention of HIV transmissions. Recently, nation-level recommendations have been made for implementation of PrEP among populations with high risk practices in Spain [68], but to date the Heath Authorities have not approved its use and reimbursement. Future implementation and scale up of PrEP or other preventive interventions may modulate the relative impact of early ART initiation.

In summary, Spanish recommendations for early ART initiation regardless of CD4 count are likely to be cost-effective and could avert 30% of new HIV cases in Spain. Improving the HIV care continuum including primarily diagnosis, and retention in care will further amplify this impact, and such a combined approach to HIV prevention should be considered a healthcare priority for Spain.

## Supporting information

S1 File (DOCX)
