# Supplementary material for: Economic and epidemiologic impact of guidelines for early ART initiation irrespective of CD4 count in Spain
Source: PLoS One. 2018 Nov 5;13(11):e0206755. doi: 10.1371/journal.pone.0206755 (PMC6218062; doi:10.1371/journal.pone.0206755)
Supplement: S1 File — (DOCX) [file pone.0206755.s001.docx]

S1 File: Supplementary Material

**Economic and Epidemiologic Impact of Guidelines for Early ART initiation irrespective of CD4 count in Spain**

Parastu KASAIE^1^, Matthew RADFORD^3^, Sunaina KAPOOR^2^, Younghee JUNG^1^, Beatriz HERNANDEZ NOVOA^3^, David W DOWDY^1^, Maunank SHAH^2*^

*Principal Investigator

**Affiliations:**

1) Johns Hopkins Bloomberg School of Public Health

2) Johns Hopkins School of Medicine

3) ViiV Healthcare Inc

**Corresponding author:** Parastu Kasaie ([pkasaie@jhu.edu)](mailto:pkasaie@jhu.edu)), 615 N. Wolfe Street, Room E6530, Baltimore, Maryland 21205

# simulation design and calibration

## Model description

The Johns Hopkins HIV Economic - Epidemiologic Model (JHEEM) is a deterministic model of linear differential equations (published previously)^[1, 2]^ that was updated to represent the Spanish HIV epidemic, and subdivides the Spanish population into risk groups. In this analysis, people living with HIV (PLWH) were categorized into groups with high – risk practices if they were heterosexual youth males and females (i.e. 15 - 30 years old), men who have sex with men (MSM) (age - stratified) or people who inject drugs (PWID), and low - risk groups if they were older heterosexual males and females (30 - 83 years old) as shown in Figure A.


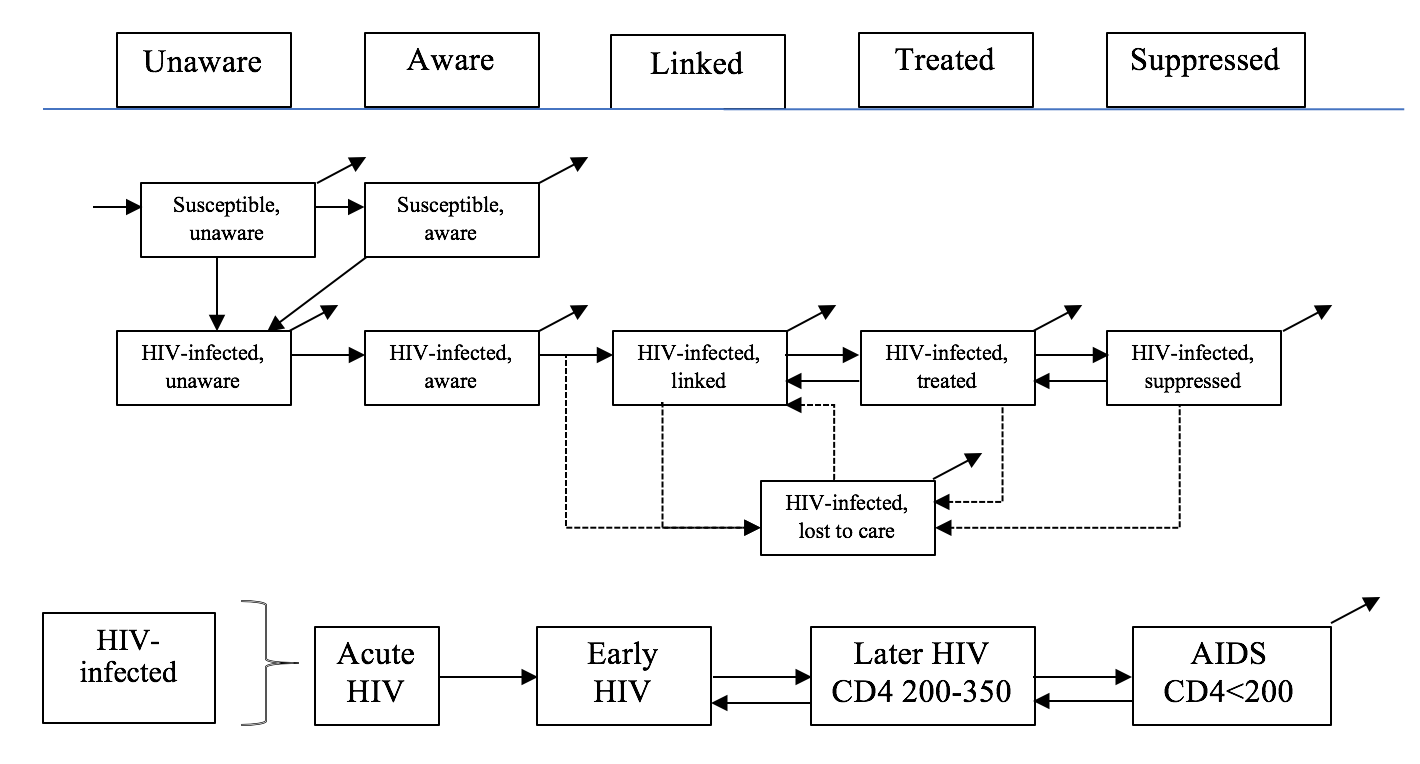


Figure A: Model schematic^[3]^. Arrows that do not lead to another box represent death or leaving the model.

Model parameters pertaining to the probabilities of average transmission per partnership, frequency of partnerships and rates of care engagement (based on gender, sexual behavior, stage of HIV infection, ART usage and awareness of HIV serostatus) were calibrated using a Bayesian melding approach (see section B). HIV transmission was modeled via heterosexual contact, male same-sex contact and needle-sharing. The HIV care continuum was modeled through HIV diagnosis among PLWH who were unaware of their HIV status. Newly diagnosed individuals were considered linked to care if they adhered to an HIV clinic visit within one month of diagnosis, but they were also eligible to engage into care later. The ART treatment was offered to individuals at CD4 count less than 350, as per guidelines for ‘delayed ART’ during the model calibration period. In the intervention simulations, ‘early ART’ initiation was modeled immediately after engagement in care (see section C). Individuals taking ART became virologically suppressed and experienced immunological recovery, with concomitant reductions in transmissibility and improved clinical outcomes^[4-11]^. We modeled ART adherence as annual risk of virologic failure, but assumed availability of alternative suppressive treatment regimens for those in care. Individuals not retained in care experienced viremia or loss of virologic suppression with subsequent impact on transmissibility. Furthermore, our model incorporates AIDS events and non-AIDS events and includes non-opportunistic-infection related HIV death for those not on ART, as well as increased mortality rates after achieving AIDS diagnosis; these event rates are reduced with ART usage according to current literature ^[9]^.

We performed all analyses using R version 3.2.4 (R Foundation for Statistical Computing). Differential equations were solved using the R package deSolve with a time step of 1 month. Further information regarding model structure and underlying system of differential equations are provided as a part of supplementary material in the original publication ^[3]^.

## Model Parameters

For full listing of parameter values, please see previously published supplemental materials^[1, 2]^. Simulation parameters fall into two categories, including fixed and variable parameters. Fixed parameters are those for which strong individual-level evidence from the literature exists (Table 1 of the main Manuscript); these parameters are set to specific values throughout the modeling process (though varied in sensitivity analysis), and include parameters describing costs, quality-adjusted weights, event rates, etc. Variable parameters are those for which an individual-level estimate is unknown or unavailable, including parameters describing patterns of mixing and HIV transmission. These parameters are calibrated in an iterative stepwise procedure designed to create a simulated population that matches selected population-level targets (as described in the calibration section below). Table A provides a list of additional model parameters and the corresponding ranges used in calibration procedure.

Table A: List of additional model parameters and associated values at baseline. Groups with risk practices include male & female young heterosexuals, young & old MSM, male & female PWIDs, and male & female old heterosexuals.

| Description | | Fix value / |
| --- | --- | --- |
|  | | variable range^[[1]](#footnote-1)^* |
| Deaths | |  |
| Rate of death once CD4<200 | | [0.2 - 0.5] |
| Relative risk reduction in mortality once on suppressive ART | | [0.03 - 0.07] |
| Marginal excess death rate for PWID per year | | 0.00711 |
| Marginal excess death rate for HIV infection off ART | | [0.001 - 0.003] |
| Screening | |  |
| Annual Rate of HIV screening, by risk group | | [0.01 - 0.15] |
| Annual additional testing frequency based on symptomatic presentation at higher CD4 counts | | [0 - 0.02] |
| Rate of additional annual testing once CD4<200 as a result of symptomatic presentation | | [0.1 - 1] |
| Sensitivity of a standard 4th generation HIV test | | 0.995 |
| Specificity of a standard 4th generation HIV test | | 0.995 |
| Linkage | |  |
| Rate of linkage irrespective of risk group after diagnosis for those with AIDS | | 0.9 |
| Proportion linking to care after diagnosis within the first 3 months, by risk practice group | | [0.3 – 0.9] |
| Retaining in care | |  |
| Rate of disengagement in care annually | | [0.05 - 0.15] |
| Relative risk for disengagement in care, by risk practice group | | [0.75 - 1.5] |
| Rate of people that were previously linked and default coming back into care | | [0.25 - 1.5] |
| **Transmission** | |  |
| Number of needle sharing partners per year^[[2]](#footnote-2)^ | | [1 - 20] |
| Proportion of MSM that engage in injection drug use | | [0.01 - 0.05] |
| Proportion of sexual partners that come from within the risk practice group | | [0.6 - 0.9] |
| Number of female partners per year among MSM | | [0.05 - 0.25] |
| Frequency of annual partnerships for older heterosexual men/women | | [0.5 - 4] |
| Frequency of annual partnerships for young heterosexual men | | [1.5 - 6] |
| Frequency of annual partnerships for young heterosexual women | | [3.5 - 6] |
| Frequency of annual partnerships for young MSM | | [3 - 8] |
| Frequency of annual partnerships for older MSM | | [3 - 8] |
| Frequency of annual partnerships for male/female PWID | | [1 - 3] |
| Probability of transmission per needle partnership | | [0.002 - 0.004] |
| Probability of transmission per sexual partnership if partner is HIV infected, by risk practice group | | [0.02 - 0.07] |
| Relative increase in probability of transmission if partner has acute HIV | | 12 |
| Relative risk reduction in probability of transmission per partnership once on suppressive ART | | [0.03 - 0.07] |
|  |  |  |

# Bayesian calibration procedure

In the primary analysis, we used a Bayesian estimation approach for calibrating the simulation model and making future projections as adopted by the Joint United Nations Programme on HIV/AIDS for HIV epidemic projections ^[12-14]^. In this approach, rather than relying on a single ‘best-fit’ parameter set, we evaluate a large number of plausible simulations based on the uncertainty ranges of epidemiologic estimates of HIV incidence, prevalence and death between 2010 and 2015 (calibration targets) - Table B.

For this purpose, we first identified a large (>10,000) sample of randomly generated simulations at equilibrium to match the distribution of PLWH in Spain in year 2010 (estimated at ~ 145,000 [120000 - 170000]) ^[15-17]^. We used these simulations to project the initial compartment sizes at year 2010. Estimating the prior distributions of unknown transmission and care-engagement parameters in the model, we next performed a large (>25000) sample of random simulations (initialized at year 2010 according to PLWH size distribution in the initial step), and ran the model forward for 5 years (2011 to 2015) to identify parameter sets that yielded epidemiologic outputs consistent with the observed HIV epidemiology during this time period in Spain. Comparing the simulated and reported levels of HIV incidence and deaths during this timeline, we then generated some measures of “likelihood” for weighting the simulations according to the goodness of fit to calibration targets. During the calibration period, we assumed minimal to no early ART initiation, consistent with national guidelines. Given the existing uncertainty in reported value of epidemiological targets in consecutive years, we only used representative values of HIV incidence and deaths at the beginning and end of calibration period (in years 2011 and 2015) as main calibration targets. Finally, we resampled the plausible simulations (>1million replications) according to assigned calibrations weights, and used the final sample to project posterior distributions for main simulation outcomes such as HIV incidence and prevalence over the next decades.

**Table B: list of epidemiological targets for the Spanish model** ^[18]^.

| Target | Year | | | | | |
| --- | --- | --- | --- | --- | --- | --- |
|  | 2010 | 2011 | 2012 | 2013 | 2014 | 2015 |
| PLWH | 145,579 | 146,378 | 146,808 | 147,231 | 147,839 | 148,879 |
| HIV incidence | 3,774 | 3,532 | 3,778 | 4,097 | 4,139 | 3,428 |
| HIV deaths | 1,266 | 1,299 | 1,297 | 1,289 | 1,273 | 1,247 |

Figure B shows the median of all generated simulations over time, as well the quartile ranges around estimated outcomes in each year. Figure C represent individual trajectories of HIV incidence and HIV deaths among the best 20 simulation projections (shown in grey) and provide a measure of variability in the underlying simulations that are used for projecting the outcomes. These results demonstrate that the JHEEM calibration accurately recapitulates HIV epidemiology in Spain.


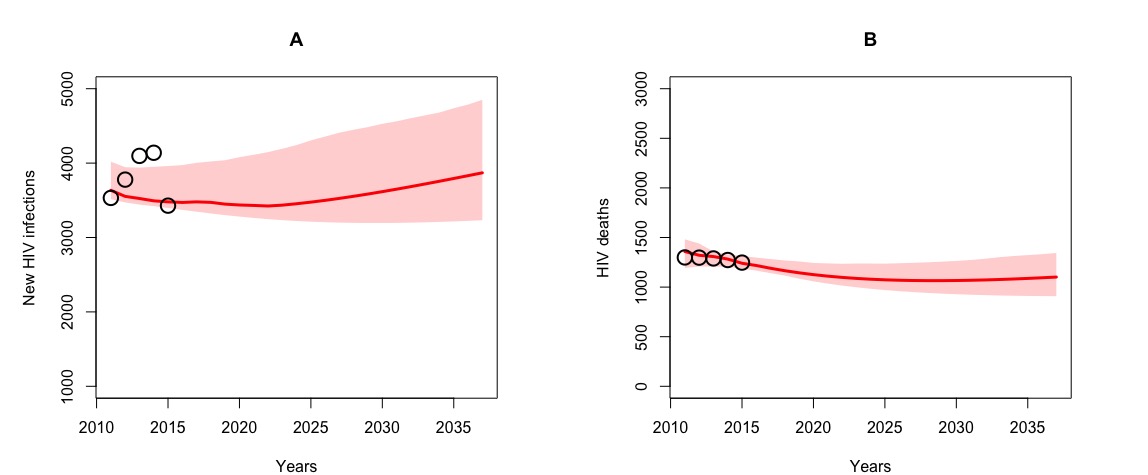
Figure B: Simulation projected levels of HIV incidence (A) and deaths (B) against calibration targets. The red line represents the median of all simulated values and shaded areas represent the quartile ranges in each year. The black marks show the available epidemiological data on HIV incidence and deaths in Spain. Given the existing uncertainty in estimates of HIV incidence from 2012 to 2014, we chose incidence in year 2011 and 2015 as the main calibration targets.


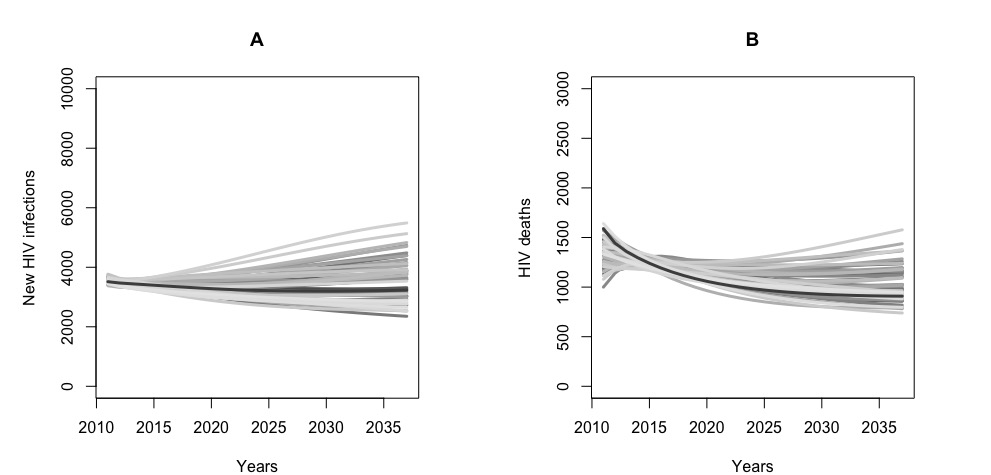


**Figure C: Simulation projected levels of HIV incidence (A) and deaths (B) among the best 20 calibrated runs.** The grey lines show individual simulation runs in the order of fitness to calibration targets in year 2011 and 2015 (dark grey showing the best run).

# Intervention scenrios

Table C shows a list of intervention scenarios modeling early ART initiation in combination with improvements to various steps of the care cascade. For a complete list of model parameters and their original values in the baseline model, see Table B.

Table C: List of modeling scenarios and associated parameter values.

| Scenario | Description |
| --- | --- |
| Early ART | Modeling ART initiation for all individuals regardless of CD4 count |
| EARLY ART+ Improved screening (among high risk populations) | Modeling early ART initiation along with increased rate of HIV screening among individuals with high-risk practices including young heterosexuals, MSM and PWID (screened on an annual basis) |
| Early ART + Improved screening & Linkage | Modeling early ART initiation along with annual HIV screening among population with high-risk practices and 100% linkage to care upon diagnosis |
| Early ART + Improved screening & Linkage & Retaining in care | Modeling early ART initiation, annual HIV screening among population with high-risk practices, 100% linkage to care, and improved retention in HIV care (5% annual loss from care or 95% annual rate of retention in care with an intervention cost of [€0 – €1000] person/year) |

# results

## Baseline results

Table D compares the projected epidemiological impact and cost related outcomes for each intervention scenarios. The incremental cost - effectiveness ratios (ICERs) are computed in comparison to the delayed ART scenario.

**Table D: Costs and effectiveness of early ART initiation in Spain over 20 years**. All outcomes represent the median [95% uncertainty ranges] across simulations.

| Scenario | HIV incidence | AIDS and non-AIDS Events | HIV Deaths | Health System Costs (billions) | QALY’s | ICER |
| --- | --- | --- | --- | --- | --- | --- |
| Delayed ART (CD4<350) | 71400 [50800 – 257700] | 60000 [47200 – 131400] | 21800 [15300 – 57800] | €19.55 [€17.19 – €23.81] |  |  |
| Early ART (regardless of CD4 count) | 51500 [33700 – 181100] | 50600 [39400 – 107400] | 18000 [12200 – 47300] | €20.63 [€18.16 – €25.15] |  |  |
| Incremental (vs. delayed ART) | 20100 [83000 – 11100] | 9100 [6300 – 26400] | 3800 [11400 – 2100] | €1.05 [€0.66 – €1.63] | 33800 [21700 – 92900] | €29700 [€13700 – €41200] |
| Early ART+ Improved Testing | 29800 [16900 – 91200] | 35800 [28700 – 69500] | 7900 [4900 – 23000] | €22.96 [€20.56 – €27.41] |  |  |
| Incremental (vs. delayed ART) | 41600 [172200 – 23200] | 23100 [15700 – 67800] | 13200 [37600 – 8000] | €3.38 [€2.64 – €4.28] | 113900 [71700 – 297500] | €29500 [€12800 – €40600] |
| Early ART+ Improved Testing & linkage | 28600 [16200 – 84600] | 35000 [28000 – 66200] | 7600 [4700 – 21500] | €22.99 [€20.69 – €27.42] |  |  |
| Incremental (vs. delayed ART) | 42900 [179400 – 24300] | 23900 [16600 – 71000] | 13500 [39100 – 8400] | €3.44 [€2.67 – €4.36] | 118700 [79700 – 313000] | €28700 [€12200 – €38700] |
| Early ART+ Improved Testing & linkage & Retaining | 23200 [13400 – 62500] | 32000 [26400 – 50600] | 6000 [3800 – 14100] | €24.58 [€22.85 – €29.23] |  |  |
| Incremental (vs. delayed ART) | 47800 [197100 – 30200] | 27700 [18200 – 83000] | 15800 [45100 – 9900] | €4.94 [€3.37 – €7.45] | 134600 [95000 – 366700] | €34400 [€15600 – €48200] |

## Longer time horizon

Given the suggested increase in life expectancy of PLWH who are receiving ART at an earlier stage of disease, and expected impact of these additional life years lived on cost - effectiveness of early ART scenarios in long term, we conducted additional analyses examining longer time horizons using the primary Bayesian calibration approach. These results demonstrated increasing benefits and reduced incremental costs over time, attributable to averted HIV infections and deaths (and subsequent averted costs) over time. Results examining the projected outcomes of early ART initiation versus delayed ART initiation over 30 and 40 years are shown in Table E and Figure D.

Table E: Costs and effectiveness of early ART initiation in Spain over 30 and 40 years. All outcomes represent the median [95% uncertainty ranges] across simulations.

| Scenario | HIV incidence | AIDS and non-AIDS Events | HIV Deaths | Health System Costs (billions) | QALY’s | ICER |
| --- | --- | --- | --- | --- | --- | --- |
| 30 years: |  |  |  |  |  |  |
| Delayed ART (CD4<350) | 104900  [77800 - 193100] | 90100  [72200 - 137800] | 29900  [16800 - 59000] | €26.33  [€22.41 - €29.13] |  |  |
| Early ART | 73100  [51000 - 136100] | 75200  [55500 - 115100] | 23200  [11500 - 48400] | €27.31  [€23.15 - €29.85] |  |  |
| Incremental | 33600  [62600 - 16800] | 16300  [10500 - 27700] | 6300  [12200 - 3400] | €0.84  [€0.4 - €1.31] | 65500  [42300 - 110900] | 65500  [42300 - 110900] |
| 40 years: |  |  |  |  |  |  |
| Delayed ART (CD4<350) | 148100  [102700 - 267400] | 122200  [95500 - 193100] | 45300  [24000 - 89500] | €30.73  [€25.51 - €35.81] | |  |
| Early ART | 98600  [62900 - 188300] | 96800  [69100 - 153300] | 33400  [16100 - 70400] | €31.32  [€26.45 - €35.77] | |  |
| Incremental | 44500  [100300 - 22700] | 24200  [16000 - 46400] | 9900  [20600 - 5900] | €0.52  [€-0.67 - €1.1] | 103600  [68400 - 191500] | 65500  [42300 - 110900] |


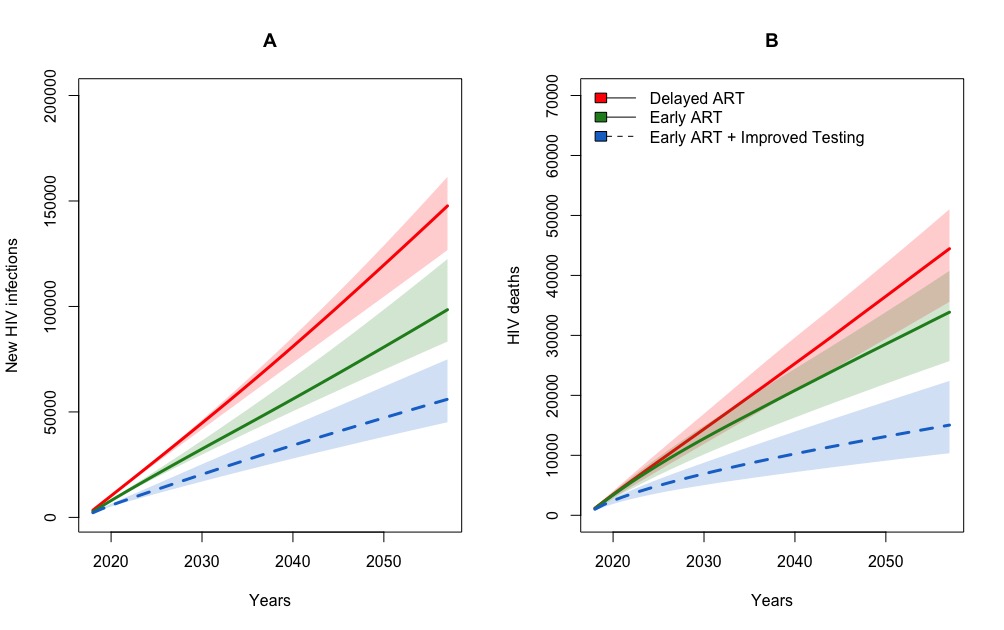


Figure D: Projected cumulative new HIV infections (A), and HIV deaths (B) in Spain from 2018 to 2057 under early versus delayed ART. All lines represent the median values of simulations, comparing scenarios representing delayed ART initiation at CD4 counts of <350 cells/mm^3^(solid red line in Panels A&C) against early ART initiation, both without (solid green line) and with additional improvements to the HIV testing (dashed blue line). The shaded areas represent the corresponding interquartile uncertainty ranges.

# Sensitivity analysis

We performed one - way sensitivity analysis by mapping the influence of each parameters (comparing the simulations containing the highest and lowest deciles of each parameter value) on additional simulation outcomes- Figure E.


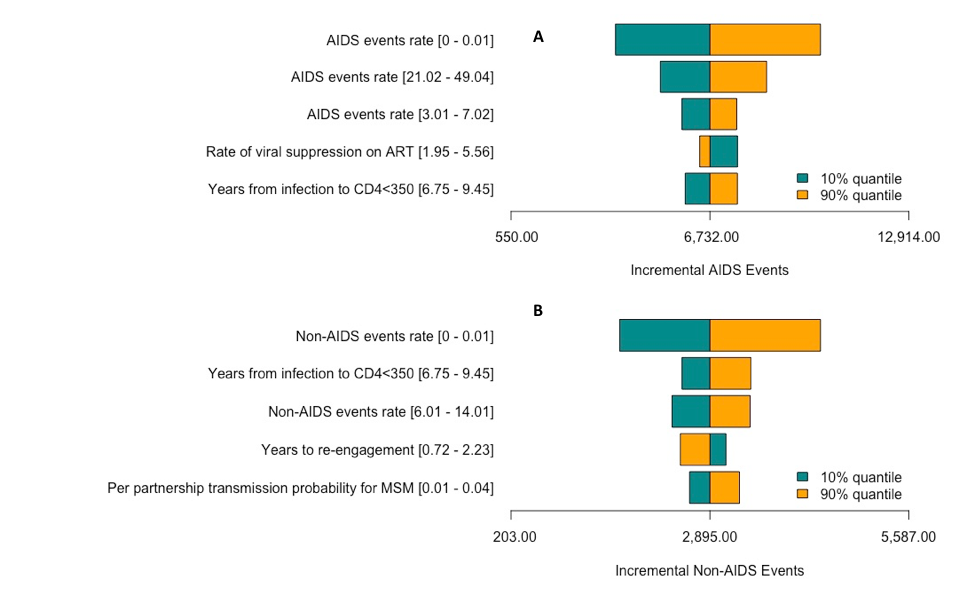


Figure E: One - Way Sensitivity Analysis of the incremental impact of early ART initiation compared to delayed ART initiation (CD4<350) to variation of all model parameters. Shown on the y - axis is the range of values for incremental AIDS-related events (A) and non-AIDS related events (B) in the early ART initiation scenario compared to delayed ART scenario. Shown on the y - axis is the list parameters [range of values] with the highest impact on each outcome. The bars represent the range of variation in selected outcomes in response to variation in value of each parameter, marked as yellow and green to represent upper and lower 10% range of all simulation results.

# Secondary Analysis with Alternative Modeling approach: Manual calibration methodology

In a secondary analysis, we used an optimization estimation approach that sought to calibrate the model to the base - case point estimates of incidence, prevalence, and death as described in surveillance reports from Spain. This approach contrasts from the Bayesian approach in the primary analysis in producing only a single ‘best - fit’ calibration parameter set, and single set of outputs for the delayed ART and early ART scenarios. We used this scenario as a reference for characterizing the model behavior under different assumptions and comparing against the Bayesian - calibrated model for further verifying the calibration procedure. Figure F shows the results of model outputs for incidence, death, and prevalence during the calibration period. Overall, the results of this alternative approach were similar to the primary analyses.


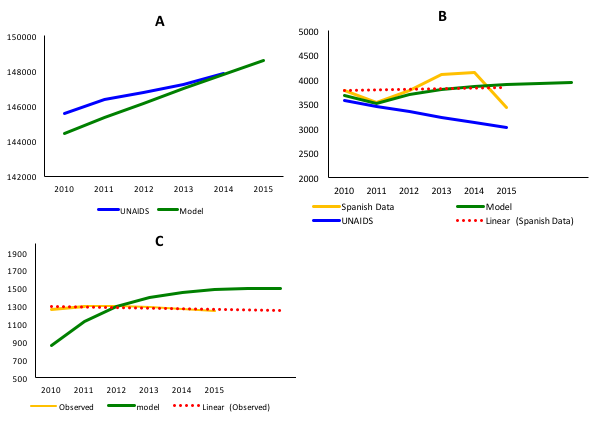


Figure F: Manual calibration results with for PLWH (A), HIV incidence (B), and HIV deaths (C). “Linear” refers to best fit lines

In the counter - factual scenario that ART initiation was continued to be delayed until a CD4 count of 350 cells/cm3, we estimated over 85,000 incident HIV cases over the next two decades in Spain, which is comparable to the average of 71,449 [50,768-257,674] incident cases projected in the primary analysis using a Bayesian approach. We projected to avert 21,500 new cases (25% reduction) with early ART (compared to projected 28% reduction in the Bayesian approach).

When examined over longer time horizons, we found that these epidemiologic impacts are amplified. We estimated 37,300 (28% reduction) and 54,700 (31% reduction) incremental cases averted over a 30 or 40 year time horizon, respectively. Overall, despite these projected benefits, our results suggest only modest improvements in the percent of PLWH that are ultimately virally suppressed (8% improvement) early ART initiation at current levels of care engagement in Spain.

If early ART was coupled with individuals with high - risk practices (younger heterosexuals, PWID, MSM) receiving an annual HIV screening test, we projected a nearly 54% reduction in new HIV transmissions (46,600 cases) compared to delayed ART initiation (39% reduction compared to early ART initiation alone) over a 20-year time horizon.

We found that improving linkage to care (at current rates of HIV screening) had modest impact on the effect of early ART, and averted 23,000 cases (21,500 with early ART alone). By contrast, if one screened individuals with high - risk practices annually and ensured that a high percentage (i.e. 100%) linked to care within 3 months, our model projected 48,000 averted cases compared to delayed ART (55% reduction) with no improvements in the care continuum. Improving all steps of the care continuum (along with early ART), was projected to have the greatest impact with 65000 cases averted (75% reduction) compared to delayed ART (with continuation of the current care continuum).

We estimated that early ART initiation would result in an incremental increase of €842 million (3% discounting of future costs and QALYs), at in incremental cost-effectiveness ratio (ICER) of €28,400 per QALY gained (95% UR 10,000 - 45,000). The incremental costs and ICER decline when projected over longer time horizons. We estimated an incremental cost of €733 million, and ICER of €11,000 per QALY gained over the next 30 years for early ART initiation; the incremental cost declines further to €370million over 40 years, at more favorable ICER’s of €3200 per QALY gained.

In secondary analysis, we found that providing annual testing to high - risk groups with early ART initiation would be more costly than delayed ART (without expanded testing), at a health system cost of €3.02billion (inclusive of annual test costs, but not of ancillary programmatic costs to reach high - risk groups), but at similar cost - effectiveness ratios given additional health benefits and averted transmissions (€28,200 per QALY gained). In one - way sensitivity analysis, we observed similar patterns of output sensitivity to variation in value of model parameters. We also found that the ICER was most impacted by the cost of ART and ranged from €18,500 to €43,000 per QALY - gained for annual ART costs ranging €6000 to €12,500 per year.

# References

1. Shah M, Perry A, Risher K, Kapoor S, Grey J, Sharma A, et al. **Effect of the US National HIV/AIDS Strategy targets for improved HIV care engagement: a modelling study**. *Lancet HIV* 2016; 3(3):e140-146.

2. Shah M, Risher K, Berry SA, Dowdy DW. **The Epidemiologic and Economic Impact of Improving HIV Testing, Linkage, and Retention in Care in the United States**. *Clin Infect Dis* 2016; 62(2):220-229.

3. Shah M, Risher K, Berry SA, Dowdy DW. **The epidemiologic and economic impact of improving HIV testing, linkage, and retention in care in the United States**. *Clinical infectious diseases : an official publication of the Infectious Diseases Society of America* 2015.

4. Hanna DB, Buchacz K, Gebo KA, Hessol NA, Horberg MA, Jacobson LP, et al. **Trends and disparities in antiretroviral therapy initiation and virologic suppression among newly treatment-eligible HIV-infected individuals in North America, 2001-2009**. *Clinical infectious diseases : an official publication of the Infectious Diseases Society of America* 2013; 56(8):1174-1182.

5. Phillips AN, Staszewski S, Weber R, Kirk O, Francioli P, Miller V, et al. **HIV viral load response to antiretroviral therapy according to the baseline CD4 cell count and viral load**. *Jama* 2001; 286(20):2560-2567.

6. Wejse C, Gomes VF, Rabna P, Gustafson P, Aaby P, Lisse IM, et al. **Vitamin D as supplementary treatment for tuberculosis: a double-blind, randomized, placebo-controlled trial**. *Am J Respir Crit Care Med* 2009; 179(9):843-850.

7. Gras L, Kesselring AM, Griffin JT, van Sighem AI, Fraser C, Ghani AC, et al. **CD4 cell counts of 800 cells/mm3 or greater after 7 years of highly active antiretroviral therapy are feasible in most patients starting with 350 cells/mm3 or greater**. *J Acquir Immune Defic Syndr* 2007; 45(2):183-192.

8. Giantin V, Perissinotto E, Franchin A, Baccaglini K, Attanasio F, Maselli M, et al. **Ambulatory blood pressure monitoring in elderly patients with chronic atrial fibrillation: is it absolutely contraindicated or a useful tool in clinical practice and research?** *Hypertension research : official journal of the Japanese Society of Hypertension* 2013; 36(10):889-894.

9. Group ISS, Lundgren JD, Babiker AG, Gordin F, Emery S, Grund B, et al. **Initiation of Antiretroviral Therapy in Early Asymptomatic HIV Infection**. *The New England journal of medicine* 2015; 373(9):795-807.

10. Cohen MS, Chen YQ, McCauley M, Gamble T, Hosseinipour MC, Kumarasamy N, et al. **Antiretroviral Therapy for the Prevention of HIV-1 Transmission**. *The New England journal of medicine* 2016; 375(9):830-839.

11. Cohen MS, Chen YQ, McCauley M, Gamble T, Hosseinipour MC, Kumarasamy N, et al. **Prevention of HIV-1 Infection with Early Antiretroviral Therapy**. *New England Journal of Medicine* 2011; 365(6):493-505.

12. Alkema L, Raftery AE, Brown T. **Bayesian melding for estimating uncertainty in national HIV prevalence estimates**. *Sex Transm Infect* 2008; 84 Suppl 1:i11-i16.

13. Alkema L, Raftery AE, Clark SJ. **Probabilistic Projections of Hiv Prevalence Using Bayesian Melding**. *Ann Appl Stat* 2007; 1(1):229-248.

14. Brown T, Bao L, Raftery AE, Salomon JA, Baggaley RF, Stover J, et al. **Modelling HIV epidemics in the antiretroviral era: the UNAIDS Estimation and Projection package 2009**. *Sex Transm Infect* 2010; 86 Suppl 2:ii3-10.

15. UNAIDS. **AIDSinfo**. In; 2016.

16. IGUALDAD MDSSSE. **Plan Estratégico de Prevención y Control de la infección por el VIH y otras infeccionesde transmisión sexual 2013-2016**. In; 2017.

17. España: ÁdVdVyCdRVEdVyse, Sida. SdIsNDdVyRNdCd. **Plan Nacional sobre el Sida - S.G. de Promoción de la Salud y Epidemiología / Centro Nacional de Epidemiología - ISCIII. Madrid; Nov 2016.** In; 2017.

18. UNIADS. **Spain-Country progress report** In; 2016.

1. * The variable ranges are used for calibration procedure as discussed in Section B. [↑](#footnote-ref-1)
2. Female partnership numbers are calculated to balance all sexual partnerships. [↑](#footnote-ref-2)
